# Short- and Long-Term Effects of Interleukin-2 Treatment on the Sensitivity of Periadolescent Female Mice to Interleukin-2 and Dopamine Uptake Inhibitor

**DOI:** 10.1371/journal.pone.0064473

**Published:** 2013-05-24

**Authors:** James S. Rankin, Steven S. Zalcman, Youhua Zhu, Allan Siegel

**Affiliations:** 1 Department of Psychiatry, New Jersey Medical School, University of Medicine and Dentistry of New Jersey, Newark, New Jersey, United States of America; 2 Department of Neurology and Neurosciences, New Jersey Medical School, University of Medicine and Dentistry of New Jersey, Newark, New Jersey, United States of America; University of Fukui, Faculty of Medical Sciences, Japan

## Abstract

Interleukin (IL)-2, a T-helper 1 (Th1) cell-derived cytokine, which potently modulates dopamine activity and neuronal excitability in mesolimbic structures, is linked with pathological outcomes (e.g., schizophrenia, depression, etc.) that at least partly reflect alterations in central dopaminergic processes. It has been suggested that dopamine neurons undergo pruning during adolescence and abnormalities in pruning predispose individuals to behavioral disorders. Since IL-2 is known as a neurodevelopmental factor affecting associated behavioral processes, the present study tested whether IL-2 can modulate stereotypic behaviors in both the periadolescent and adult periods. This study determined whether IL-2 treatment would produce long-lasting changes in sensitivity to a later challenge with IL-2 or GBR 12909, a highly selective dopamine uptake inhibitor. Four experiments were conducted. Firstly, a decrease in novelty-induced stereotypic behavior was observed in BALB/c periadolescent mice (38 days of age) following IL-2 administration (0.4 µg/2 ml) relative to vehicle control. In the second experiment, an initial dose of IL-2 was given in the periadolescent period, but did not affect rearing responses. A second dose of IL-2 given to the animals 30 days later as adults, resulted in a significant increase in rearing behaviors relative to control animals. In the third experiment, separate groups of experimental and control mice were administered GBR 12909, a highly selective dopamine reuptake inhibitor, 30 days following treatment with either IL-2 or vehicle. It was noted that this experimental group, which initially received IL-2, exhibited stereotypy, as evidenced by increased sniffing behavior. A fourth experiment revealed that IL-2 administered in periadolesecence and adulthood had no effect on other motor responses, indicating that IL-2 selectively modulates selective stereotypic behaviors. The results provide evidence, for the first time, that long-term changes in stereotypy in periadolescent mice are linked to an IL-2 mechanism, possibly utilizing dopamine.

## Introduction

Exposure to stress during critical neurodevelopmental periods can induce long-term effects on brain and behavior. For example, maternal stressor exposure in rats leads to abnormalities in the thickness of the cerebral cortex, while exposure to stress during the perinatal period can lead to long-term consequences on behavior and reproductive capacity [1]. The majority of the literature investigating long-term behavioral consequences of stressor exposure during developmental periods focuses on prenatal and early life periods, but very little is known about how stressor exposure during adolescence affects adult behavioral response to challenges during adulthood.

During adolescence, individuals undergo a process known as “synaptic pruning”, or a loss of the excess connections between neurons overproduced early in development [2]–[4]. One implication of this “synaptic pruning” is aberrant pruning incited by immune or environmental factors during critical windows in development, which has been associated in the development of schizophrenia, among other neuropsychological conditions [3], [5]. Attention deficit disorder (ADD), another important developmental aberration, relates to dopaminergic cells, with alterations in pruning of dopaminergic synapses and receptors during adolescence. As such, periadolescent rodents (30–45 days of age) that are concurrently experiencing pruning have been thought of as a model for several behavioral disorders in human populations; these include schizophrenia and drug-abuse, both of which become apparent during an analogous time period in human adolescence [6]–[8]. Evidence from various lines of research suggests that stressors and immune-derived stimuli during critical developmental periods may have profound short- and long-term consequences on behavior. However, little is currently known about the mediators in this process.

Recent data suggests that infection or inflammation, like psychogenic or physical stressors, during the course of gestation may predispose individuals to pathologies of the meso-limbic and -cortical dopamine systems, such as an increased expression of stereotypic behavior (excessive functional motor behaviors), deficits in learning and hypersensitivity to drugs of abuse [9], [10]. It is generally thought that cytokines released during the immune response play an important role in mediating such effects. Concerning IL-2, it is a cytokine produced by T-helper cells involved in immune response, which has been shown to directly alter dopaminergic activity in the meso-limbic and -striatal pathways, thereby altering behaviors associated with these systems. This leads to speculation as to the interplay between stressors, IL-2 and behavioral alteration exacted through the dopaminergic pathway.

IL-2 modulates dopamine levels during pre- and post-natal periods, as well as in adulthood, and dopamine-mediated behaviors during adulthood. Given that a major developmental period for dopaminergic neurons occurs during adolescence (i.e., when the pruning process occurs), IL-2 has also been shown to alter development of dopaminergic systems. For example, De Araujo *et al.* and McAfoose *et al.* showed that IL-2 increases the survival and synaptogenesis of dopaminergic neurons during development [11], [12]. In parallel with this finding, it has been posited that cytokines, in their regulation of synaptic plasticity, may be causative agents of neuropsychiatric syndromes.

There is a dearth of information, however, on the effects of cytokines on the adolescent brain and its maturation. The effect of immune mediators (e.g. IL-2) during a window period of vulnerability, such as neural restructuring during adolescence, on these systems is therefore of great interest. Accordingly, the present study tested the hypothesis that IL-2 modulates stereotypy when administered in periadolescent mice and such effects are linked to a dopamine mechanism. This hypothesis was examined by determining the behavioral effects that IL-2 exerts on periadolescent female mice in an initial challenge and to a subsequent challenge in adulthood with IL-2 or GBR-12909, a dopamine reuptake inhibitor, which may also function as a psychostimulant.

## Materials and Methods

### Subjects

All methods and procedures were approved by Institutional Animal Care and Use Committee (IACUC) of UMDNJ-NJMS, Newark, NJ. A total of 36 female BALB/c mice (Charles River Laboratories, Wilmington, MA), 38 days of age (mean initial weight of 18.2 g ±0.33 g) were bred for use in this study. This strain of mouse was selected based on our previous investigations from our laboratory in the examination of neurochemical and behavioral changes and age was selected based on what is defined as adolescence in mice [8], [13]. The animals were housed individually in polypropylene ‘shoebox’ cages, maintained on a 12-h light/12-h dark cycle (7 am–7 pm) and permitted ad libitum access to food and water. Experiments were conducted during the light phase.

### Reagents

Recombinant, murine, and carrier-free IL-2 (PeproTech Inc., Rock Hill, NJ) was dissolved in sterile saline and injected subcutaneously (s.c.) at doses of 0.4 µg/mouse in approximately 2 mL. Dose and route of administration were chosen based on our demonstration that exploration was significantly increased in this strain of adult male mouse following a single s.c. injection of 0.4 µg of IL-2 [14].

GBR 12909 (1-{2-[bis(4-fluorophenyl)-methoxy]ethyl}-4-(3-phenylpropyl)piperazine) (Tocris-Cookson Inc., Ballwin, MO), a potent and highly selective inhibitor of dopamine uptake, was dissolved in sterile saline and injected intraperitoneally (i.p.) at a dose of 7 mg/kg. Peripheral or central injections of GBR 12909 induce behavior-activating effects, and this dopamine agonist shares properties that are associated with psychomotor stimulants that also inhibit dopamine uptake.

### Behavioral Testing

Animals were tested in a cage outfitted with a TruScan Activity Monitoring System (Coulbourn Instruments). The TruScan measured various data points, namely: Ambulatory Distance–The total of all horizontal movements minus the total distance of stereotypic moves. Jumps–The total number of time, the subject loses contact with the horizontal floor plate, not exceeding 2 seconds. Counterclockwise Turns (CCW)–counterclockwise turns into 4 sequential quadrants in the center of the cage. Vertical Plane (VP) Moves–Total movement in the vertical plane. VP Entries–The total # of times any part of the animal entered the vertical plane. VP Time–The total time any part of the animal spent in the vertical plane. VP Stereotypy (STPY) Moves–The total # of coordinate changes in the vertical dimension, <0.75 cm spaces apart in the horizontal dimension, and back to the starting point, not exceeding 2 seconds apart. Three such movements must be made before recording starts; thereafter, the 3 movements are included in the total # of moves. When the subject moves outside of the region, or does not move within them for 2 seconds, recording stops and resets. VP STPY Time–The total time of all of the episodes of stereotypic episodes defined in VP STPY Moves. Center Time–The total time spent outside of a 1.9 cm margin of the walls. Center Distance–The total of all X–Y coordinate changes occurring outside of a 1.9 cm margin of the walls. Margin Time/Distance–Center, substituted with “within a 1.9 cm margin”. Center vs. Margin (C vs. M) Time–The ratio of Center Time/Margin Time. C vs. M Distance–The ratio of Center Distance/Margin Distance.

Test sessions were also taped with a VHS camera and scored at a later date. Scoring was measured with a stopwatch for time (Sec) spent: rearing, rearing against the wall, grooming and sniffing; with less than 3 sec being recorded for number of episodes and 3 sec or more recording the time duration, except for in the instance of total numbers where all instances were counted. Use of a stopwatch was based on our demonstration that IL-2 induced significantly comparable effects on this behavior in experiments using a programmed computer or a stopwatch, while 3 sec as a threshold for time duration was chosen based on our previous experiments showing the effects of IL-2. A rearing episode was defined as a subject lifting both front limbs off the ground, whereas a rearing episode against the wall consisted of lifting both front limbs and leaning against the cage wall. We selected these stereotypic measures based on our findings that IL-2 treatment induces an increase in stereotypic motor behavior, showing that those behaviors may be attributed to elements of an activated immune system and their utility as animal analogous of repetitive stereotypic movements [15].

In experiment 1, the mice (n = 18/group) received a single subcutaneous (s.c.) injection of IL-2 0.4 µg or saline 300 µL and immediately thereafter were individually placed into a test cage for 2 hours. Measurements were taken in 2-minute epochs once every 20 minutes.

In experiment 2, we determined if periadolescent IL-2 treatment would proactively influence behavioral responses to a later injection of IL-2 (i.e. 1-month later). Accordingly, 12 mice received saline and 12 mice received IL-2 (0.4 µg, s.c.). Immediately following these injections, the mice were individually placed into the test cage, and behavior was determined, as described.

In experiment 3, we determined if periadolescent IL-2 treatment would proactively influence behavioral responses to a later injection of GBR 12909 (i.e. 1-month later). Accordingly, in Experiment 3, six mice received saline and six mice received a single intraperitoneal (i.p.) injection of GBR (7 mg/kg, i.p.).

Immediately after each injection, the animals were individually placed into their respective activity box for 2 hours, where they were recorded on TruScan and filmed and scored, at a later date, as described. Illustrated data for this experiment represent activity scores for entire 2-hour sessions.

### Statistics

Data was analyzed with one-way ANOVA’s (i.e. Experimental vs. Control group) - and two-way ANOVA’s (i.e., variable A: experimental versus control group, and B: effects over time irrespective of group) testing selective stereotypic motor and related responses using Excel software (Microsoft Co., Redmond, Wa.).

## Results

### Effects of IL-2 on Novelty-induced Behavioral Activity

**Figure 1:**
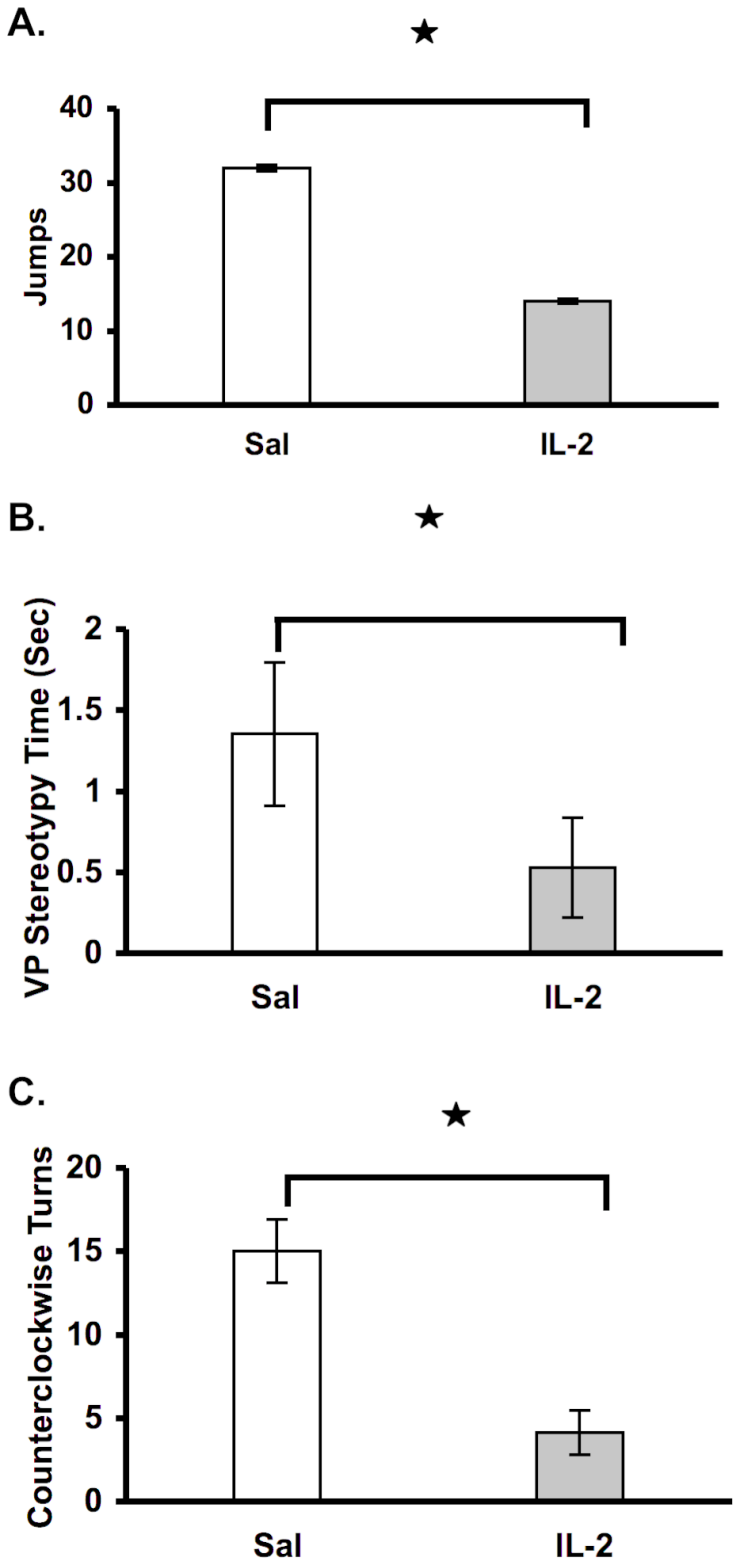
Single injection of IL-2 in periadolescent mice decreases stereotypic behaviors. Mean (± S.E.M.) activity scores (A. Jumps; B. VP STPY Time; C. CCW Turns) following a single injection of saline or IL-2 (0.4 µg/mouse, s.c.). Mice were exposed to the test cage immediately following cytokine administration. *p<0.05.

In this experiment, the goal was to determine the effects of IL-2 administration upon motor responses, which included: VP stereotypy time (VP STPY Time), turns, ambulatory distance and center vs. margin time. The results indicated a significant decrease in novelty-induced exploratory behavior as evidenced by reductions in: VP STPY Time [F1, 32 = 6.56, p<0.05] (Fig. 1a.), Jumps [F1, 32 = 6.92, P<0.05] (Fig. 1b.); and CCW turns [F1, 33 = 5.82, p<0.05] (Fig. 1c.). Interestingly, there was no significant effect on locomotion (i.e. Ambulatory distance [F1, 33 = 1.38, p = 0.25], or location in the test cage (C vs. M Time [F1, 33 = 0.52, p = 0.48] and C vs. M Distance [F1, 33 = 1.77, p = 0.19]) (figure not shown).

### Effects of Subsequent IL-2 Challenge on Rearing Behavior

In this experiment, the goal was to determine if a subsequent challenge to IL-2 administered one month later would exert a different effect on behavior, which included various modes of rearing behavior, jumps, turns and VP STPY Time.

**Figure 2:**
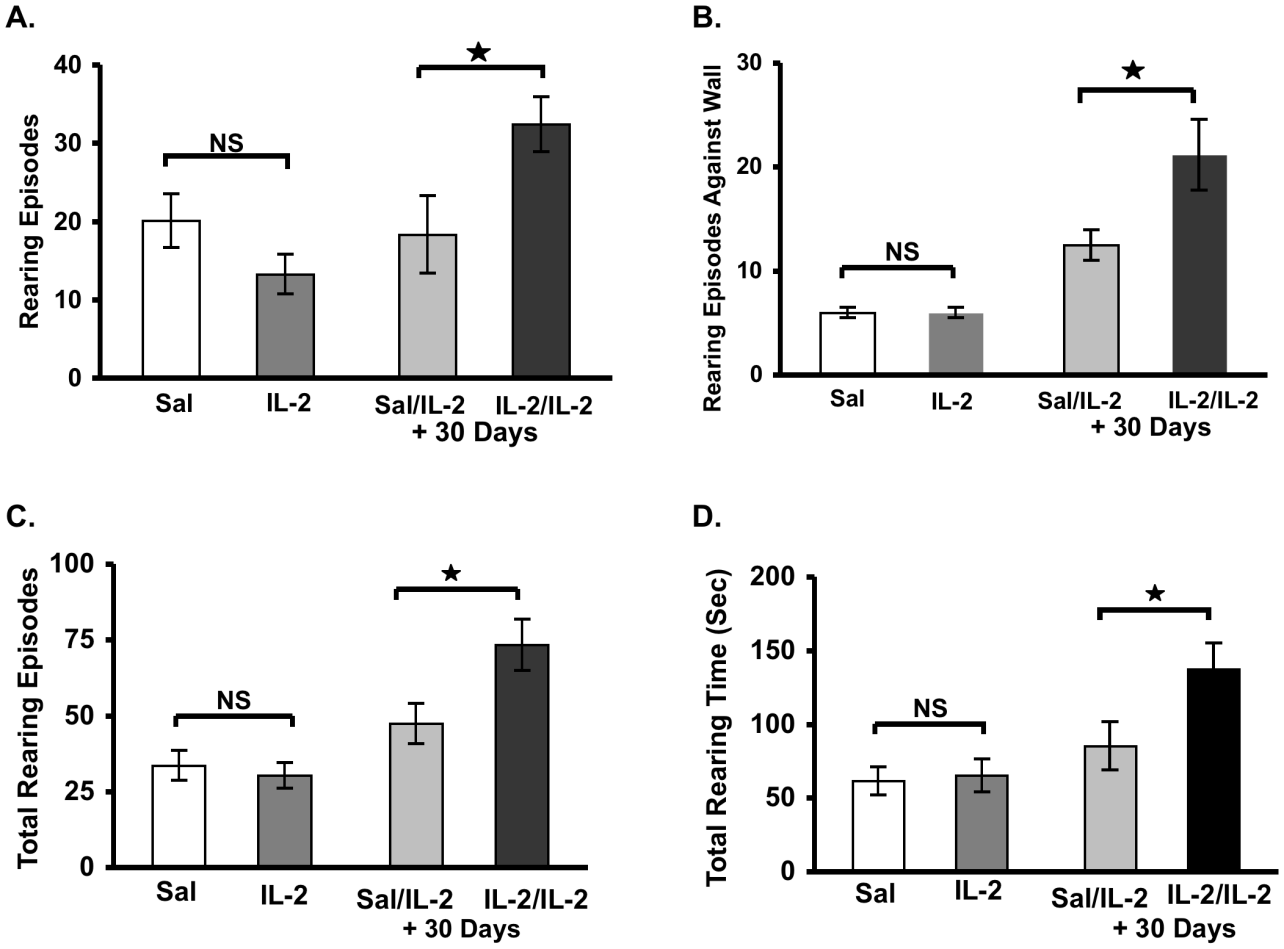
Administration of IL-2 in adults increases rearing behavior (in contrast to periadolesence). Mean (± S.E.M.) activity scores (A. Number of rearing episodes within 3 sec; B. Number of rearing against wall episodes within 3 sec; C. Total number of rearing episodes; D. Total time spent rearing (sec)) following a single injection of saline or IL-2 (0.4 µg/mouse, s.c.) and those tested 30 days later with single injection of IL-2 (0.4 µg/mouse, s.c.) administered 30 days later, as described in the figure with “+30 days” designation. Mice were exposed to the test cage immediately following cytokine administration. *p<0.05.

Administration of IL-2 during the initial (periadolescent) period had no effect on rearing behavior: Number of Rearing Episodes (less than 3 sec) [F1, 33 = 0.27, p = 0.11] (Fig. 2a.); Number of Rearing Against Wall Episodes (less than 3 sec) [F1, 15 = 0, p = 1] (Fig. 2b.); Total Rearing Number [F1, 33 = 0.28, p = 0.59] (Fig. 2c.); and Total Rearing Time (Sec) [F1, 33 = 0.08, p = 0.78] (Fig. 2d.).

When tested one month later after a subsequent challenge with IL-2, the results indicated significant increases in novelty-induced exploratory behavior, as evidenced by increases in: Number of Rearing Episodes (less than 3 sec) [F1, 18 = 4.96, P<0.05] (Fig. 2a.); Number of Rearing Episodes Against Wall (less than 3 sec) [F1, 18 = 10.19, p<0.005] (Fig. 2b.); Total Number of Rearing Episodes [F1, 19 = 5.66, p<0.05] (Fig. 2c.); and Total Rearing Time (Sec) [F1, 18 = 5.34, p<0.05] (Fig. 2d.).

Of further importance, the stereotypic behaviors that were significantly altered in mice receiving a single injection of IL-2 during periadolescence were not significantly altered by a second IL-2 injection, including: Jumps [F1, 20 = 1.46, p = 0.5]; CCW turns [F1, 22 = 0.64, p = 0.43]; and VP STPY Time [F1, 21 = 0.63, p = 0.44] (not shown).

### Effects of Selective Dopamine Uptake Inhibitor on Sniffing Behavior

**Figure 3:**
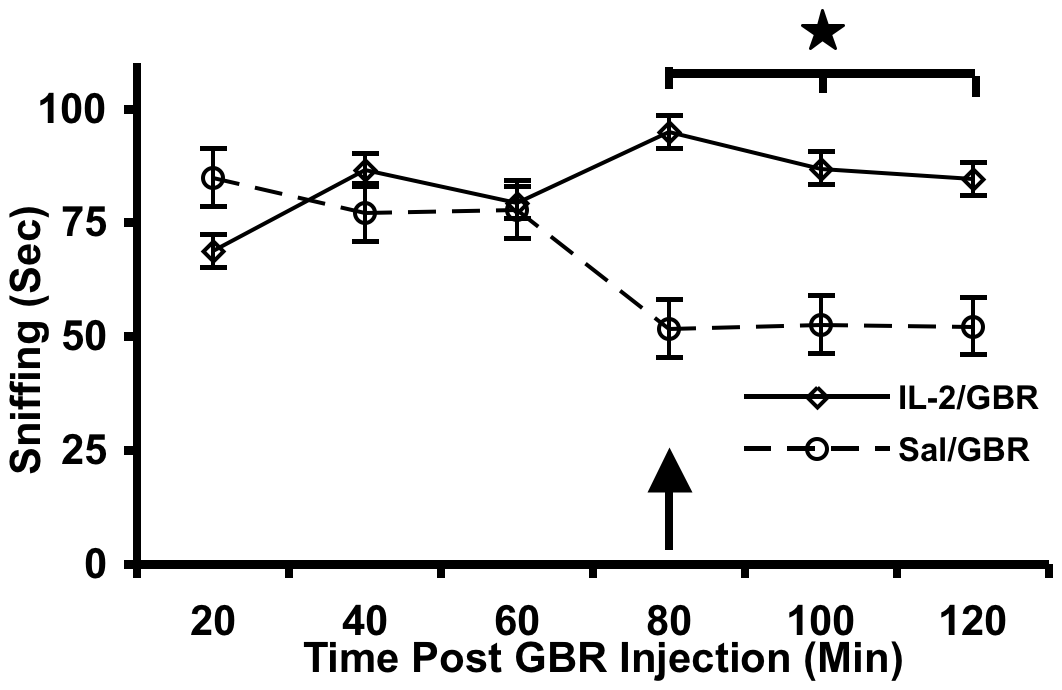
IL-2 administration in periadolescent mice increases sensitivity to GBR-induced stereotypic sniffing administered 30-days later in adulthood. Mean (± S.E.M.) activity score of GBR 12909-stimulated sniffing time. Mice received a single injection of saline or IL-2 (0.4 µg/mouse, s.c.), and were tested 30 days later with GBR 12909 (7 mg/kg, i.p.). Mice were exposed to the test cage immediately following GBR administration. Note that the effect was initially observed at 80-minutes post-injection (shown with arrow). *p<0.05.

The objective of the third experiment was to determine whether administration of GBR 12909, a highly selective dopamine uptake inhibitor, would exert a different effect on treatment versus vehicle. The results indicated a significant increase in sensitivity upon the behaviorial-stimulating effects of GBR 12909. In particular, compared with mice that received only saline during periadolescence and GBR 12909 one-month later, mice that received IL-2 during periadolescence plus GBR 12909 one-month later showed significant increases in sniffing. A two-way Treatment×Time Point after GBR 12909 ANOVA revealed a significant main effect of Treatment [F1, 5 = 5.17, p<0.05] (Fig. 3). IL-2 administration did not alter sniffing responses prior to administration of GBR. Moreover, activity, rearing, as well as other measures of stereotypic behaviors, were not significantly altered after GBR administration (not shown).

### Absence of Non-specific Motor Effects of IL-2

**Figure 4:**
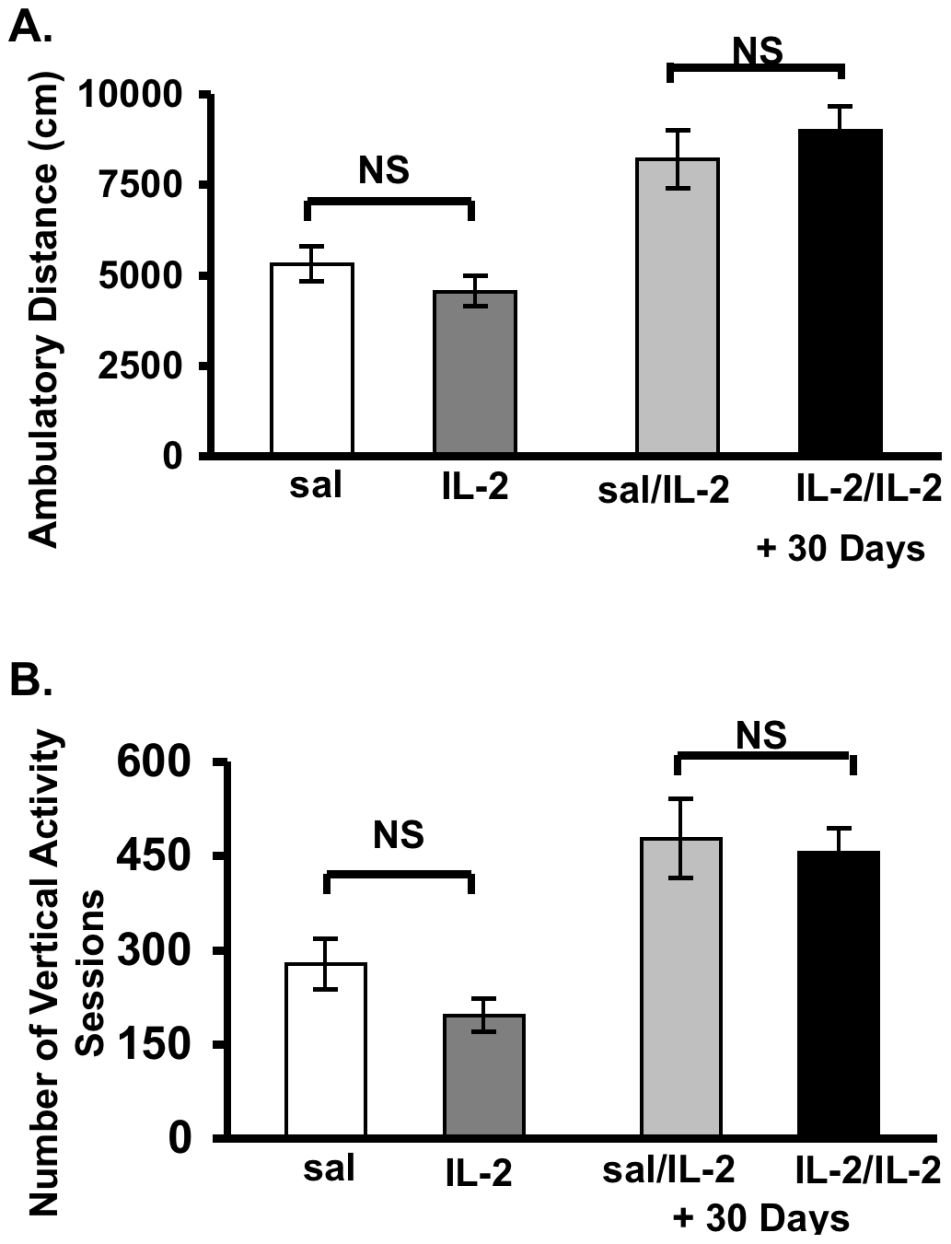
Administration of a single injection of IL-2 in periadolescent mice does not alter other motor responses (i.e. ambulatory distance or vertical activity), in contrast to the modulatory effects upon rearing shown in Figure 2. A second injection of IL-2 30-days later also did not alter these responses. Mean (± S.E.M.) activity scores ((A). Ambulatory distance (cm); (B). Number of vertical activity sessions) following a single injection of saline or IL-2 (0.4 µg/mouse, s.c.) and those tested 30 days later with single injection of IL-2 (0.4 µg/mouse, s.c.), as described in figure with “+30 days” designation. p>0.05 NS [NS = non-significance].

In order to determine whether or not the results observed in the experiments shown above were due to non-specific effects of IL-administration, the following data were compared and shown in Figs. 4a and 4b, as a means of displaying the specificity of the behaviors exhibited. Both ambulatory distance and vertical plane activity were not significantly different between groups receiving initial administration of IL-2 versus saline ([F1, 33 = 1.38, p = 0.2] and [F1, 33 = 2.7, p = 0.11], respectively). Likewise, when the same groups both received IL-2 one-month later, there were no significant differences in these motor responses tested ([F1, 22 = 1.47, p = 0.24] and [F1, 22 = 0.66, p = 0.55], respectively). Therefore, this finding indicates that IL-2 had a selective effect upon the specific motor responses whose results were described above in this study.

## Discussion

In the present study, we showed for the first time that cytokine administration induces significant and long-lasting behavioral variations in periadolescent mice. Specifically, a single injection of IL-2 induced significant decreases in novelty-induced stereotypic behavior (Figs 1 and 2), possibly indicating a “depressive-like” state, which did not significantly change overall activity in the horizontal or vertical directions (Fig. 4). This finding therefore reveals an absence of non-specific motor effects of IL-2 upon stereotypic behaviors tested in this study. Of further importance, adult responses to IL-2 or psychostimulant challenge (i.e., GBR 12909) were significantly enhanced in mice with a history of IL-2 treatment during periadolescence. Thus, a single injection of IL-2 during the periadolescent period has important consequences on adult responses. This suggests a temporal specificity upon selective behavioral motor responses that are associated with specific ages of the animals examined in this study. In addition, based upon the recent findings of Zalcman *et al.*, it is also likely that these effects are mediated through specific anatomical loci and their associated pathways [15].

Of central importance is the question of whether IL-2 can influence behavioral processes via their influence on brain function. The neurochemical and behavioral effects of peripherally applied IL-2 are related to its ability to cross the blood-brain-barrier (BBB), but its mode of entry is unclear. In adults, the molecular weights of cytokines are sufficiently large to prevent them from crossing the BBB alone to activate neurons by specific receptor mechanisms or to produce corticotropin-releasing hormone (CRH). In adolescents, the blood brain barrier (BBB) may be more porous and underdeveloped and therefore, pro-inflammatory cytokines may enter through the “leakier” area, act on brain regions lacking one of these barriers or they may enter the brain via “specific uptake systems” [16]–[18]. Under conditions when IL-2 is able to cross the BBB, it has been shown to influence dopamine turnover in the prefrontal cortex and dopamine release in the nucleus accumbens [19]. There is also evidence for entrance of T_h_1-Lymphocytes directly into the CNS across the BBB in experimental allergic encephalomyelitis (EAE) and other experimental models, which may be affected by inflammatory states [20], [21].

### Factors Affecting Stereotypic behavior in Periadolescent Mice

Compared with mice that received saline during the periadolescent period, young mice with a history of IL-2 treatment showed significant increases in rearing behavior following a single injection of IL-2 during young adulthood. Notably, IL-2 did not induce this effect when administered during periadolescence; whereupon the following conclusions can be made: (1) The individual’s initial response to IL-2 was not sensitized upon subsequent exposure to the cytokine; and (2) one cannot predict the animal’s behavioral response to a second injection of IL-2 based on its initial behavioral responses to the cytokine. We suggest that changes in relevant neural pathways (e.g., meso-limbic and –striatal systems) induced by IL-2 administration could serve as better predictors.

### Dopamine

The possibility that IL-2 acts through a neurochemical mechanism in the brain has recently been established, in which a specific form of aggressive behavior (i.e., defensive rage) is powerfully modulated by IL-2 acting through GABA_A_ receptors in the medial hypothalamus and by substance P- neurokinin (NK_1_) receptors in the midbrain periaqueductal gray [22], [23]. Therefore, from the findings of the present study, it is reasonable to suggest that IL-2′s effects upon stereotypy are also mediated through a neurotransmitter mechanism, namely via dopamine receptors. It was observed that GBR 12909-induced increases in stereotypic behavior in adult female mice were potentiated in mice that received a single injection of IL-2 in periadolescence, implying that IL-2 produced long-lasting changes in the sensitivity of meso-limbic and –striatal dopaminergic activity. That responses to IL-2 or GBR 12909 were further augmented in mice with a history of IL-2 treatment during adolescence implies that an increase in IL-2 during that period has profound effects on the sensitivity, and perhaps development, of relevant neural systems.

The relationship between dopamine and various forms of motivated and abnormal emotional behavior has been studied extensively. There is a positive relationship between rearing behavior, IL-2 [14], [24] and dopamine in the ventral striatum [25], which is of interest because of its relationship between motor functions and goal-seeking/motivational responses, in addition to the fact that this region of the forebrain projects to other areas involved in motor and tic disturbances [3], [15]. Therefore, it is possible that dopamine’s effects upon both motor and emotional processes are mediated in part through a cytokine mechanism.

A progressive number of studies show relationships between inflammatory processes, cytokines, and neuropsychiatric disorders in humans (i.e. depression, schizophrenia) [10], [26]–[31]. IL-2 influences monoaminergic neurotransmission in the CNS and is often linked to psychological diseases such as depression, or anxiety disorders [32]–[34]. IL-2 and soluble IL-2 receptors are elevated in the CSF of schizophrenic patients [19], where increased IL-2 may be related to positive symptoms and sIL-2R, a modulator of IL-2 levels. These levels may reflect an inhibitory process associated with IL-2 related to increases in dopaminergic transmission [35].

Inasmuch as IL-2 treatment administered during periadolescence increased sensitivity to the motor activating effects of IL-2 and GBR 12909, coupled with the fact that the initial exposure to IL-2 occurred during the periadolescent period, we suggest that IL-2 influences the pruning process during which dopaminergic synapses and receptors are eliminated. To be sure, this is highly speculative; nevertheless, IL-2 is known to affect the development of dopaminergic neurons during pre/post-natal periods [11], [12], [36]–[38].

### Anatomical Considerations

Based upon recently published work in our laboratory, there is now evidence that the behavioral effects of IL-2 upon stereotypy is mediated primarily through neurons in the neostriatum, nucleus accumbens and motor regions of cortex [15], [39]. IL-2 is synthesized centrally, where it is distributed throughout the CNS. In addition, increased c-Fos expression (suggesting increased metabolic activity) in these forebrain neurons following stereotypic behavior overlap extensively with soluble IL-2 receptor labeling in the same regions of forebrain, which suggest that these areas are associated with several of the behavioral motor processes examined in the present study [15], [24], [40]–[42].

Therefore, these observations, coupled with the findings of the present study, would suggest that these regions of the forebrain might provide the substrate for the enhanced effects of IL-2 upon stereotypy. The precise physiological mechanisms by which stereotyped behaviors are modulated by IL-2 remain to be elucidated.

### Other Possible Factors

IL-2 has been shown to alter hormone levels (i.e. CRH, ACTH and their down-stream mediators) and modulate the hypothalamic-pituitary-adrenocortical (HPA)-axis [16], [43]. Given this fact and that glucocorticoids regulate dopaminergic cell activity, it should also be considered that IL-2 induced potentiation of the HPA response played a role in long-term effects associated with this cytokine.

What is also known is that the periadolescent period is a critical time for development of the HPA-gonadal axis and its effects on behavior. As opposed to the postnatal period, which may exhibit long-term consequences for male mice, female mice exposed to stressors during adolescence may cause alterations in behavior during adulthood, which may be attributed to differential levels of microglia within the brain based on sex hormone concentration [44]. Furthermore, it has been shown that certain female sex hormones affect neurotransmitter systems in various brain regions (e.g., dopamine in the dorsal striatum) [43], [45], [46]. Although, we did not control for random hormonal cycling, and while the mice employed in the present study could have been randomly cycling females, we nevertheless observed significant effects between groups, suggesting a possible significant role played by dopamine in this process.

In summary, we provide novel evidence that a single injection of IL-2 in periadolescent female mice significantly modifies behavioral responses to produce a long-lasting increase in sensitivity to later challenges with IL-2 or GBR 12909. It remains to be seen, however, whether IL-2 in periadolescent mice modifies dopamine, the HPA axis and/or cortisol levels. We suggest that alterations in IL-2 in periadolescence increases vulnerability to the expression of psychopathological outcomes involving increased repetitive stereotypic behaviors that may be associated with abnormalities in the pruning of dopaminergic neurons and synapses during adolescence, This has significant implications on our understanding of adolescent and periadolescent behavior in that we demonstrated that administration of a pro-inflammatory agent during the periadolescent period induces significant unique effects during this time period and has long-term behavioral consequences.

Future studies would require measurement of cortisol levels to differentiate between the effects of IL-2 and GBR in modulating the stress response, which could include diminished functions of the HPA-axis or increases in dopaminergic functions. Additionally, tests would further require an analysis of the labeling patterns of c-Fos following stereotypic and related responses to localize the brain regions involved in cortisol neuronal activation, as well as direct measurement of dopamine and its receptors in response to IL-2 administration.
